# Facial disfigurement due to olfactory neuroblastoma: beauty regained with chemotherapy

**DOI:** 10.1002/cnr2.1303

**Published:** 2020-10-08

**Authors:** Parmod Kumar, Deepak Sundriyal, Rekha Bhandari, Abhijeet Singh, Bhinyaram Jat, Amit Sehrawat

**Affiliations:** ^1^ Department of Medical Oncology, Hematology All India Institute of Medical Sciences Rishikesh Uttarakhand India; ^2^ Department of Pathology All India Institute of Medical Sciences Rishikesh Uttarakhand India; ^3^ Department of Head and Neck Surgery All India Institute of Medical Sciences Rishikesh Uttarakhand India

**Keywords:** chemosensitive, neoadjuvant chemotherapy, olfactory neuroblastoma

## Abstract

**Background:**

Olfactory neuroblastoma (ONB) is a sinonasal malignancy seldom seen in clinical practice. It is also known by various other names like esthesioneuroblastoma, esthesioneuroepithelioma, esthesioneurocytoma, and esthesioneuroma. Surgery and radiation therapy are considered as standard treatment modalities for ONB; however, the role of chemotherapy is not well established.

**Aims:**

We aim to define the role of chemotherapy in the neoadjuvant setting in a case of ONB.

**Methods and Results:**

We report a young female patient presenting with a naso‐facial swelling causing facial disfigurement, proptosis, decreased visual acuity, and poor performance status. She was diagnosed with advanced‐stage ONB. Prompt administration of chemotherapy led to the improvement in the symptoms and rapid regression of the tumor mass. Later on, the tumor mass was excised completely without any neurological deficit.

**Conclusion:**

This report justifies the role of neoadjuvant chemotherapy in the management of ONB.

## 1 INTRODUCTION

Olfactory neuroblastoma (ONB) is an uncommon malignancy of neuroectodermal origin. It usually arises from the sensory neuroectodermal olfactory cells situated in the nasal cavity. The estimated incidence is 0.4 cases per million populations.^1^ The paucity of cases does not allow a prospective clinical trial in search of a standard treatment protocol. Moreover, the biology of the disease is highly variable ranging from slow‐growing indolent tumors to locally aggressive, and metastatic lesions. This leads to inconsistency in the recommended treatment protocols. Surgical resection followed by radiation therapy has been considered as the optimum treatment protocol for most of the cases of ONB; however, the role of chemotherapy is not clearly established. We report a case of aggressive ONB in a young female. The aggressive nature of the disease led to her facial disfigurement with an imminent threat to loss of vision. Expedient administration of chemotherapy led to regaining her normal facial features and restoration of normal vision.

### 1.1 Case

A 32‐year‐old female from a remote hilly village presented with an 8‐week history of progressive swelling around the nose, and severe bouts of headaches. For the last 2 weeks, she was also experiencing episodes of recurrent epistaxis, diminution of vision, and double vision. According to her husband, the disease had rapidly progressed in the last 4 weeks leading to severe facial disfigurement and now it had become difficult to recognize her. She was unable to do household chores and self‐care, being mostly confined to bed. On examination, the patient had a swelling involving the whole of the nose with facial puffiness and proptosis of the right eye. The right vestibule of the nose was full of some necrotic appearing mass. Bilateral level I and II neck nodes were enlarged. She had marked anhedonia and was chair‐bound, not responding to any queries.

**FIGURE 1:**
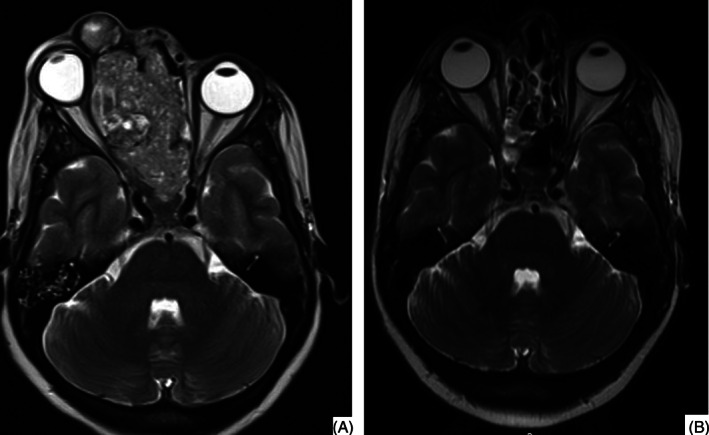
T2‐weighted images of the MRI. Panel A showing the tumor mass involving para‐nasal sinuses and causing proptosis; Panel B showing the reduction in the tumor size and resolution of proptosis after neoadjuvant chemotherapy

A contrast‐enhanced magnetic resonance imaging (MRI) of the brain and neck revealed a multi‐lobulated lesion of size 8.2 × 4 × 6.5 cm involving the right nasal cavity. The lesion was reaching anteriorly up to the external nare with infiltration into the upper lip, bilateral gingivobuccal sulcus, right masticator space, hard palate, and projecting posteriorly into the nasopharynx (Figure 1, panel A). It had completely destroyed the nasal septum, turbinates, and cribriform plates with extension into the bilateral orbit and infiltrating into the medial rectus muscle and optic nerve. It had displaced the right globe and resultant proptosis. There was evidence of invasion of frontal dura, sphenoid sinus, and pituitary gland. Imaging of the thorax and abdomen and a bone scan revealed no evidence of metastasis.

**FIGURE 2:**
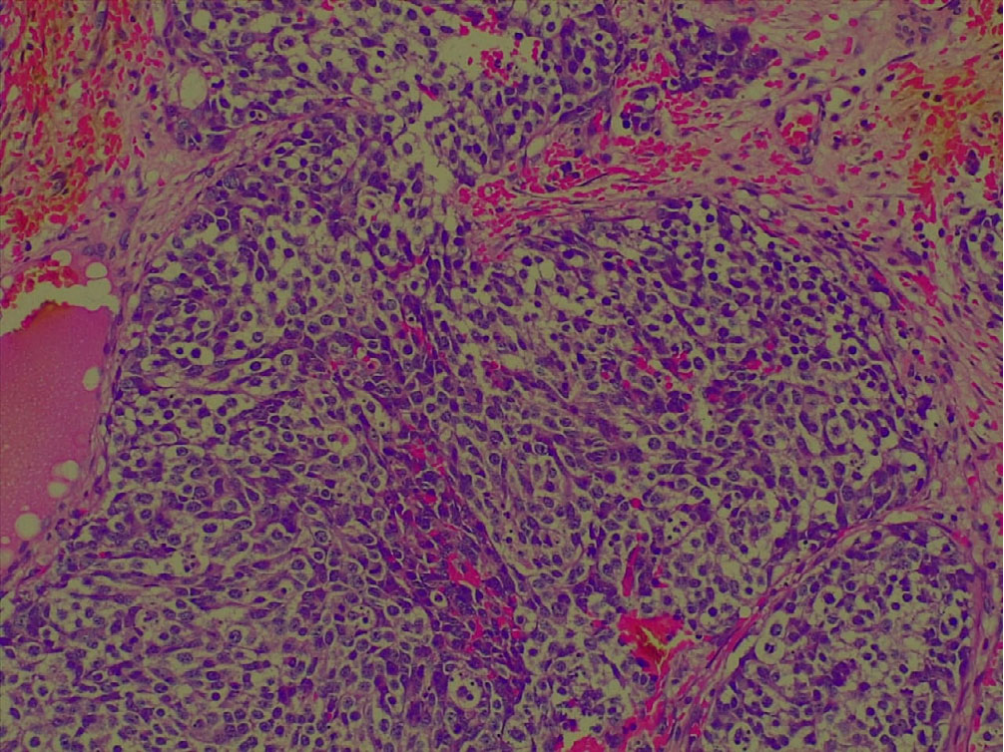
Photomicrograph showing monomorphic small round tumor cells with granular chromatin arranged in lobules and nests. (Hematoxylin & Eosin 40 x)

Her blood counts, liver, renal, coagulation, and the metabolic panel were within normal limits. A biopsy from the right nasal cavity mass was obtained and it showed a lobulated tumor composed of nests of cells with scanty to moderate cytoplasm with round to oval nuclei dispersed in a neurofibrillary stroma (Figure 2). Tumor cells were immune positive for pan‐CK and chromogranin while they were negative for p40. Ki‐67 labeling index was 70%.

A final diagnosis of ONB, Kadish stage D, Hyams grade III was reached.

Her case was discussed in the multidisciplinary tumor board. Since it was very difficult to achieve an R0 resection without much morbidity and neurological deficits, neoadjuvant chemotherapy (NACT) was planned. However, in a patient with poor performance status (PS) and severe depression, it was difficult to administer chemotherapy. The patient's husband was counseled in detail regarding the guarded prognosis and pros and cons of chemotherapy and he consented for the treatment.

**FIGURE 3:**
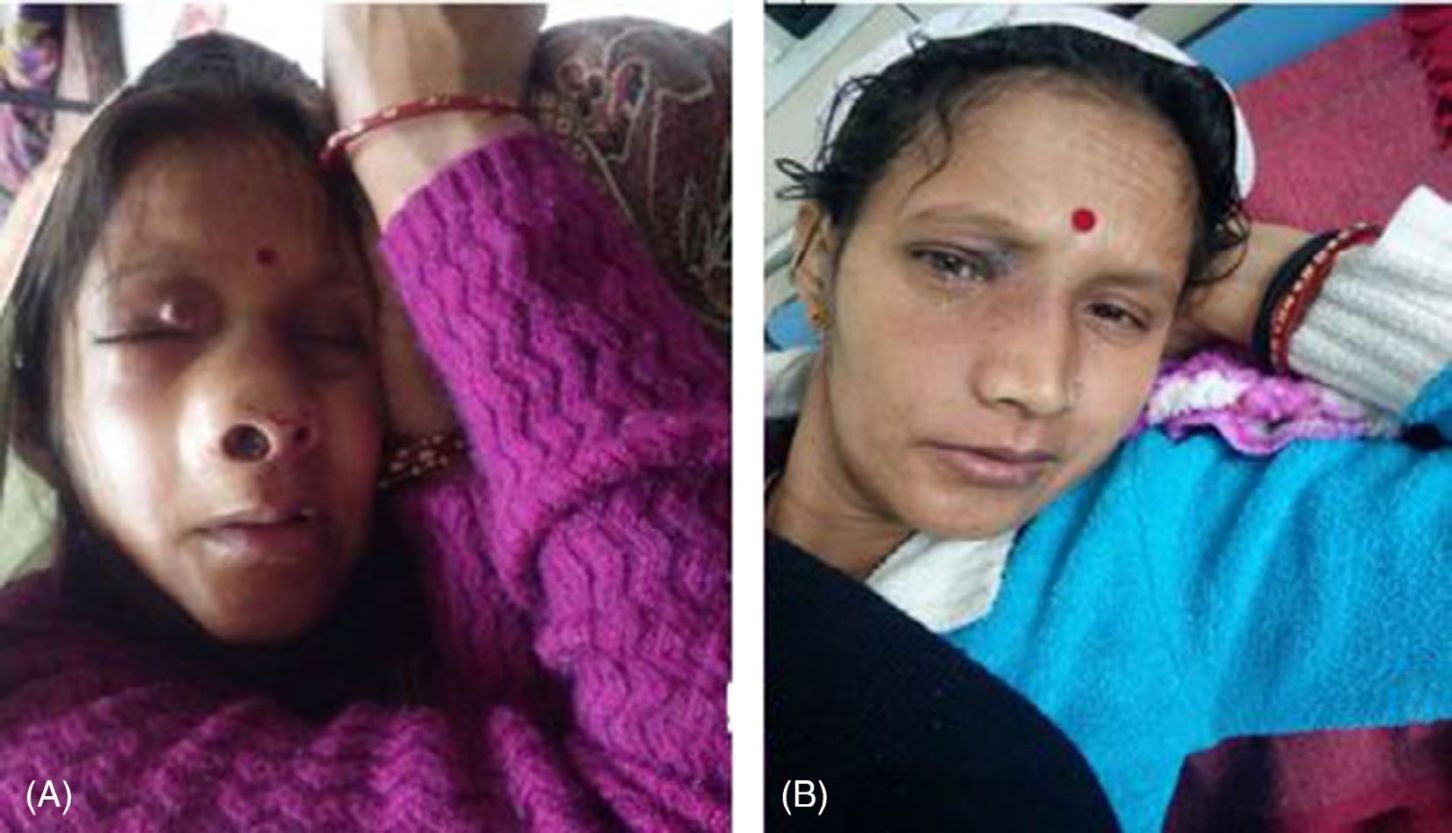
Clinical response to chemotherapy on day 14 of the first cycle. (A) Pre‐chemotherapy; (B) Post‐chemotherapy

Tumor lysis prophylaxis in the form of allopurinol was given. We administered a 3‐day protocol of cisplatin (25 mg/m^2^) and etoposide (75 mg/m^2^); however, the dose was reduced by 15% keeping in view the poor PS of the patient. There was a striking improvement in signs and symptoms. Her epistaxis and headache resolved on day 7 post‐chemotherapy. Visual acuity improved and the double vision disappeared by day 10. On day 14, facial swelling and proptosis resolved, and PS started improving (Figure 3). Depression was resolved completely by the end of the first cycle of chemotherapy.

The dose of chemotherapy was optimized and she received four cycles of the same protocol without any significant adverse effects. MRI done for response evaluation after four cycles was suggestive of a very good response to therapy (Figure 1, panel B). She was referred to the head and neck surgeon for surgical management. The patient underwent an endoscopic craniofascial resection of the tumor via modified Denker's approach. Intraoperatively, pinkish, polypoidal soft tissue mass in the right nasal cavity and b/l maxillary, ethmoid, sphenoid sinus with obliteration of frontal recess with mucocele formation was noted. Bony dehiscence was noted over ipsilateral lamina papyracea, cribriform plate, planum sphenoidale, and optic nerve and suprachiasmatic region, however, dura and periorbita were found intact. The tumor was excised in piece meal using debrider and coblation. The soft tissue adherent to the cribriform region and planum was sent for the frozen section, which was negative. The peri‐operative period was uneventful. There was no neurological deficit. Post‐operative histopathological examination of the resected tumor mass was suggestive of pathological complete response. She received two more cycles of the same protocol as the adjuvant therapy and subsequently referred to the radiation oncologist for adjuvant radiation therapy.

## 2 DISCUSSION

A clinical staging system originally defined by Kadish et al. and later modified by Morita et al. classifies ONB into four stages based on the involvement of nasal cavity, paranasal sinuses, local extension, and regional or distant nodal metastases.^2^, ^3^ Hyams histological grading system based on cytoarchitecture, mitotic rate, nuclear polymorphism, rosette formation, and necrosis defines the aggressive of the disease and prognosis.^4^

There is no specific age group of presentations for ONB and cases have been reported from extremes of ages.^5^ Defining a standard treatment protocol for ONB is onerous due to the rarity of the tumor, variant biological behavior, and resemblance with other malignancies found in the same location as sinonasal neuroendocrine carcinoma, undifferentiated carcinoma, lymphoma, and rhabdomyosarcoma.

A review of 17 studies published from 1990 until 2019 showed that surgery and adjuvant radiation therapy provided the best overall survival and progression‐free survival especially in patients with high Kadish stage. The role of chemotherapy was not clear.^6^ A recent review of 149 articles also suggested surgical resection followed by radiotherapy as the standard approach for higher‐grade tumors.^7^

A beneficial role of chemotherapy in ONB has been suggested owing to its biological behavior similar to other chemosensitive neural crest tumors like high‐grade neuroendocrine tumors.^8^ These tumors have a high Ki‐67 labeling index which is a positive predictive factor for response to chemotherapy. Goldsweig and Sundaresan suggested that ONB and neuroblastoma share a common histogenesis and both might have similar sensitivities to chemotherapeutic agents like cyclophosphamide, vincristine, and cisplatin.^9^

However, small sample sizes, variability in the chemotherapy regimen, timing (neoadjuvant vs adjuvant), and sequencing of the therapy have made it difficult to formulate a definite recommendation. A recent systemic review and meta‐analysis of advanced stage ONB suggested the role of chemotherapy in combination with surgery and/or radiation therapy. The choice of the regimen was not specified.^10^ A neoadjuvant role of chemotherapy has been suggested by a recent case series in which six patients of ONB presenting with intracranial extension were treated with NACT with cisplatin, etoposide, and ifosfamide followed by surgery and radiotherapy. The authors opined that NACT could be an effective treatment for tumor reduction, improving surgical resection, and reducing its complications.^11^ A retrospective review of 15 cases of ONB with advanced stage presenting to the MD Anderson Cancer Center revealed that patients treated with induction chemotherapy had a response rate of 68% with a response rate of 78% in the high Hyams grade group. Chemotherapy regimen used were cisplatin plus etoposide, and cyclophosphamide plus doxorubicin plus vincristine. Seven patients achieved a complete response as per the radiological criteria. They concluded that ONB is a chemosensitive tumor and induction chemotherapy is an acceptable modality of treatment for the advanced‐stage disease.^12^

The available evidence now suggests that ONB is a chemosensitive tumor and there is a definite role of chemotherapy in the management, albeit not very well defined. Chemotherapy can be used as an induction or neoadjuvant component rendering tumor more amenable to definite therapy in the form of surgery or radiotherapy. The mutilating surgery and the comorbidities after the surgery or radiation therapy can be avoided by the use of upfront chemotherapy. The impact of chemotherapy on survival, role in the adjuvant setting, and the choice of the regimen are, however, not well defined.^13^, ^14^

Our case has several salient features. The patient had a poor PS and she was in severe depression. Administration of chemotherapy under such circumstances is difficult as it may do more harm than benefit. However, a cautious decision was taken as biology and history of the tumor suggested that the tumor might be responsive to chemotherapy. The patient regained her facial features which were otherwise lost due to disfigurement caused by the expansile tumor. Her vision was restored. She achieved a PCR which has not been reported in ONB.

## 3 CONCLUSIONS

1. ONB can present like a locally aggressive tumor, and prompt evaluation and treatment is necessary to prevent disease‐related morbidities.
2. The tumor is highly sensitive to chemotherapy and NACT should be administered to downsize the tumor amenable to R0 resection.
3. A cautious trial of chemotherapy can be done in patients with highly chemosensitive tumors who are otherwise unfit for systemic anti‐cancer therapy due to poor PS or comorbidities.

### PATIENT'S PERSPECTIVE

My wife was very sick and in great depression for the last 1 month. I had lost all hopes. I was very skeptical about chemotherapy as I had many myths regarding the ill‐effects of chemotherapy. The treating team explained to me about the treatment and I showed faith in them. Her condition started improving remarkably within a few days after starting chemotherapy. Her vision has become normal. She has now completed her chemotherapy and surgery and we are thankful to the treating team.

## COMPETING INTERESTS

The authors declare that they have no competing interests.

## AVAILABILITY OF DATA AND MATERIAL

Available on request from the corresponding author.

## ETHICS STATEMENT

Approval from the institutional ethics committee and consent from the patient was obtained for the publication of this report.

## AUTHOR CONTRIBUTIONS

DS, BJ, RB, and PK collected and interpreted the data. RB, AS, AS, and PK prepared the manuscript. DS, AS, PK, AS, and BJ were involved in patient management. All authors read and approved the final manuscript.

## Data Availability

Available on request from the corresponding author
